# ClusterEnG: an interactive educational web resource for clustering and visualizing high-dimensional data

**DOI:** 10.7717/peerj-cs.155

**Published:** 2018-05-21

**Authors:** Mohith Manjunath, Yi Zhang, Yeonsung Kim, Steve H. Yeo, Omar Sobh, Nathan Russell, Christian Followell, Colleen Bushell, Umberto Ravaioli, Jun S. Song

**Affiliations:** 1 Carl R. Woese Institute for Genomic Biology, University of Illinois at Urbana-Champaign, Champaign, IL, United States of America; 2 Department of Bioengineering, University of Illinois at Urbana-Champaign, Champaign, IL, United States of America; 3 Illinois Applied Research Institute, University of Illinois at Urbana-Champaign, Champaign, IL, United States of America; 4 Department of Electrical and Computer Engineering, University of Illinois at Urbana-Champaign, Champaign, IL, United States of America; 5 Department of Physics, University of Illinois at Urbana-Champaign, Champaign, IL, United States of America

**Keywords:** Validation measures, Genomics, Web interface, Education, Clustering

## Abstract

**Background:**

Clustering is one of the most common techniques in data analysis and seeks to group together data points that are similar in some measure. Although there are many computer programs available for performing clustering, a single web resource that provides several state-of-the-art clustering methods, interactive visualizations and evaluation of clustering results is lacking.

**Methods:**

ClusterEnG (acronym for Clustering Engine for Genomics) provides a web interface for clustering data and interactive visualizations including 3D views, data selection and zoom features. Eighteen clustering validation measures are also presented to aid the user in selecting a suitable algorithm for their dataset. ClusterEnG also aims at educating the user about the similarities and differences between various clustering algorithms and provides tutorials that demonstrate potential pitfalls of each algorithm.

**Conclusions:**

The web resource will be particularly useful to scientists who are not conversant with computing but want to understand the structure of their data in an intuitive manner. The validation measures facilitate the process of choosing a suitable clustering algorithm among the available options. ClusterEnG is part of a bigger project called KnowEnG (Knowledge Engine for Genomics) and is available at http://education.knoweng.org/clustereng.

## Background

Clustering is one of the most powerful and widely used analysis techniques for discovering structure in large datasets by grouping data points that are similar according to some measure. Several programming languages such as R (R Core Team , 2015) and Python (Pedregosa et al., 2011) offer libraries or packages for clustering custom data and generating static plots. However, interactive visualization, which aids the user in understanding the data at a deeper level, requires additional libraries and external software. Moreover, the advent of next-generation sequencing has enabled researchers to generate data at an unprecedented rapid pace, creating an acute need for resources that can enable the users of high-dimensional biological data to quickly perform “first-hand” analysis, such as clustering (Stephens et al., 2015). The main challenges to building such a resource are handling large datasets and facilitating its interpretability. Client-side computer systems or web browsers may not always be powerful enough for efficient navigation through the data. The NIH has recently funded Big Data to Knowledge (BD2K) Centers to tackle this type of challenges. As part of the KnowEnG BD2K Center, we have developed a web-based resource called ClusterEnG (acronym for Clustering Engine for Genomics) for clustering large datasets with efficient parallel algorithms and software containerization.

Web servers, such as ClustVis (Metsalu & Vilo, 2015), provide a simple yet powerful interface for visualizing Principal Component Analysis (PCA) and heatmap plots. However, at present, ClustVis limits the uploaded file size to 2 MB, and the plots are also static. WebMeV (Wang et al., 2017), a cloud-based application, performs PCA, k-means and hierarchical clustering on large datasets, while providing limited interactivity and visualization. Gitools (Perez-Llamas & Lopez-Bigas, 2011) contains several features for interactive visualization of clustering results, but currently there is no web interface available. Also, at present, Gitools provides only two clustering algorithms for analysis. WebGimm (Joshi et al., 2011) is another application for clustering analysis of gene expression data and provides results to be viewed externally using various Java applications. Similarly, other existing tools, although catering to high-dimensional data, either require a local software installation or lack clustering visualization and validation analysis (L’Yi et al., 2015; Fernandez et al., 2017). In comparison, ClusterEnG integrates the features of the above tools into one platform and produces visual results with enhanced interactivity. ClusterEnG’s interactive PCA and t-Distributed Stochastic Neighbor Embedding (t-SNE; Van der Maaten & Hinton, 2008) plots in 2D and 3D allow intuitive exploration of structures in data. ClusterEnG also provides additional algorithms not available in the above resources. Furthermore, ClusterEnG offers several internal validation measures, thereby adding a crucial feature for evaluating the performance of clustering results.

## Results

**Figure 1:**
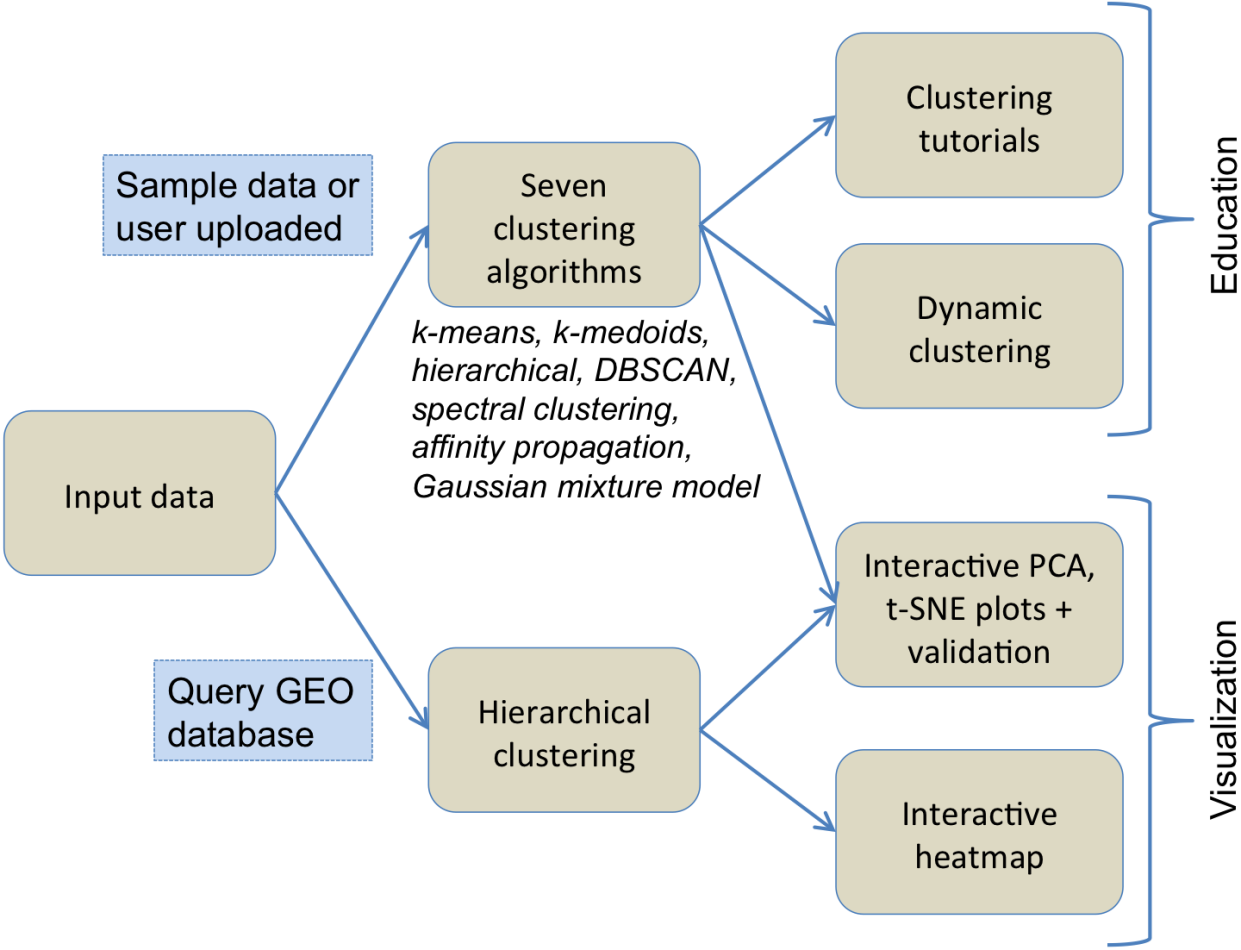
Typical workflow of ClusterEnG encompassing educational and visualization components.

Figure 1 illustrates the flowchart of various components of ClusterEnG, from user-uploaded data to output visualizations. Underlying details of the components are outlined below.

### Input data and output

The user can upload custom data or choose one of the preloaded sample datasets for clustering. ClusterEnG accepts data in a tabular format of rows and columns, allowing the user to analyze most datasets generated by typical biological experiments, such as RNA-seq, microarray and drug-response data. The input data are then read in R utilizing the fast and convenient “fread” function from the *data.table* package (Dowle et al., 2015). The ClusterEnG server currently accepts files up to a size of 2 GB. The uploaded file will be securely stored on the server temporarily for seven days, during which the user can retrieve the file or run more jobs from the same browser (with cookies enabled).

Currently, the server contains two public sample datasets: the gene expression data in NCI60 cancer cell lines (Ross et al., 2000) and B-cell lymphoma cells (Alizadeh et al., 2000). The NCI60 data (9,707 genes, 64 samples) provide a good tumor gene expression dataset to explore and assess the quality of clustering from various algorithms implemented in ClusterEnG. The B-cell lymphoma data have a similar number of samples as the NCI60 data, but contains a larger number of genes (18,432 genes, 67 samples).

Clustering results are made available to the user in a CSV format in mainly two different ways. First, the user can download a single file with the entire data, sample/feature annotation and clustering results. Second, the user can select a subset of data interactively and download the clustering labels for the chosen data points. The user can also download snapshots of clustering plots in PDF, PNG and SVG formats.

### Clustering algorithms

**Figure 2:**
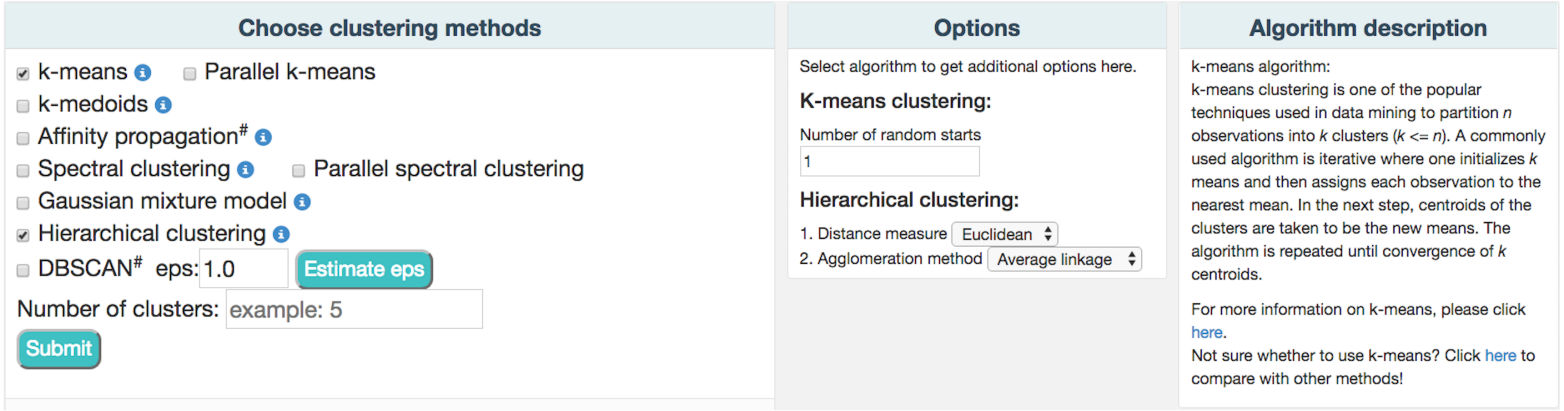
A partial snapshot of ClusterEnG user interface showing a choice of clustering algorithms and related options.

ClusterEnG provides seven clustering algorithms, including parallel implementations for two algorithms. Currently, serial implementations are written in the R programming language using various packages available in the CRAN repository (R Core Team , 2015). The seven algorithms include k-means, k-medoids, affinity propagation, spectral clustering, Gaussian mixture model, hierarchical clustering and DBSCAN (Ester et al., 1996). Two heuristic algorithms are also implemented to estimate one of the parameters of DBSCAN algorithm. The parallel code for the k-means algorithm utilizes a software package written in C (Liao, 2005), while parallel spectral clustering implements a C++ code (Chen et al., 2011). For a subset of the algorithms, the user is given a list of commonly used parameters to modify and visualize the changes (Fig. 2).

ClusterEnG also features a module for querying the Gene Expression Omnibus (GEO) database (Davis & Meltzer, 2007) to download data and draw an interactive heatmap with hierarchical clustering based on the InCHlib JavaScript library (Skuta, Bartunek & Svozil, 2014). This allows direct access to published biological data and deeper exploration of hierarchical clustering results.

### Docker containerization

We employ state-of-the-art methods to handle the analysis of large files. The input data and user-selected algorithms from the front-end are dynamically packaged into a Docker container (Merkel, 2014) on the back-end wherein the code (serial or parallel) is executed and the results are returned to the main server. Chronos is used to schedule jobs by spawning Docker containers into an Apache Mesos cluster, which automatically utilizes available processors for parallel runs.

### Interactive 2D/3D visualization

**Figure 3:**
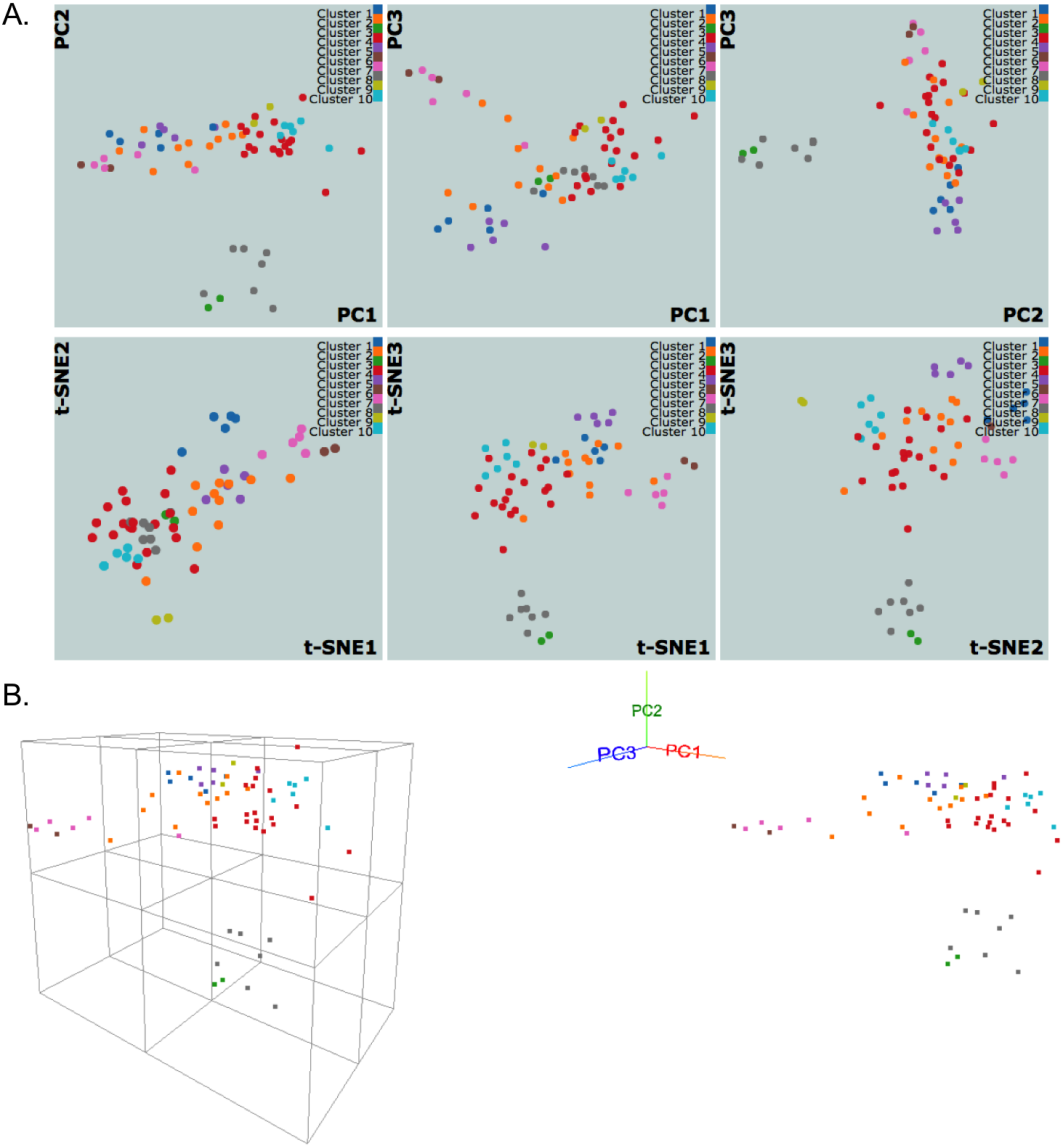
NCI60 gene expression sample data clustering of samples using k-medoids algorithm. The snapshots show visualizations of first three principal components and vectors from PCA and t-SNE, respectively, in (A) 2D and (B) 3D with perspective and orthogonal projection of principal components.

We use dimensionality reduction techniques to facilitate the meaningful visual interpretation of the clustering results. Currently, PCA and t-SNE plots, which are broadly used in diverse fields, are implemented. We utilize the R packages *stats* and *Rtsne* (Krijthe, 2015) to evaluate the PCA and t-SNE algorithms, respectively. PCA and t-SNE provide complementary views; PCA is linear and deterministic, while t-SNE is nonlinear and nondeterministic. After PCA is performed, projection coefficients onto the first three principal components are used to generate three linked scatter plots for each pair of the components (Fig. 3A). Similar scatter plots are shown for t-SNE by reducing the number of input dimensions to three (Fig. 3A). Interactive plots are displayed using JavaScript library d3.js (Bostock, Ogievetsky & Heer, 2011) and jQuery to allow zooming, group selecting, mousing-over for annotation, and highlighting a region/cluster which maps to other PC direction plots.

We also implement a dynamic 3D visualization for the first three principal components to enable deeper exploration of data structure by providing a perspective 3D view of data points. A real-time orthogonal projection from the current 3D viewpoint is also provided. Written in Javascript with the libraries d3.js (Bostock, Ogievetsky & Heer, 2011) and three.js (Cabello, 2010), the 3D Principal Component Viewer (Fig. 3B) allows zooming and rotating of the viewpoint. Graphical User Interface (GUI) is written using dat.GUI to toggle points or automate the rotation of viewpoint. It should be noted that the user’s browser and machine capabilities may limit these 2D and 3D visualizations. Our preliminary tests show that the visualizations work well for up to a few thousand data points on machines with typical hardware and modern browsers, but Google Chrome performed the best in all tests.

### Internal clustering validation measures

We include internal clustering validation measures to help evaluate the clustering results. Internal clustering validation is used to measure the goodness of clustering results without referring to any external information such as class labels (Liu et al., 2010). Eighteen indices for clustering validation are calculated using relevant functions from the R package *clusterCrit* (Desgraupes, 2016), where a short summary for each index is provided on our website.

On the ClusterEnG website, the validation measures are summarized for each index and clustering algorithm. Each index has an optimal measure (minimum or maximum value), which is used to compare clustering algorithms. A donut chart displays the number of indices for which each clustering algorithm is optimal. Also, a bar chart is shown for each index to compare the index values between clustering algorithms. The calculated validation measures are available for the user to download for subsequent analysis.

### Clustering tutorial and dynamic clustering

**Figure 4:**
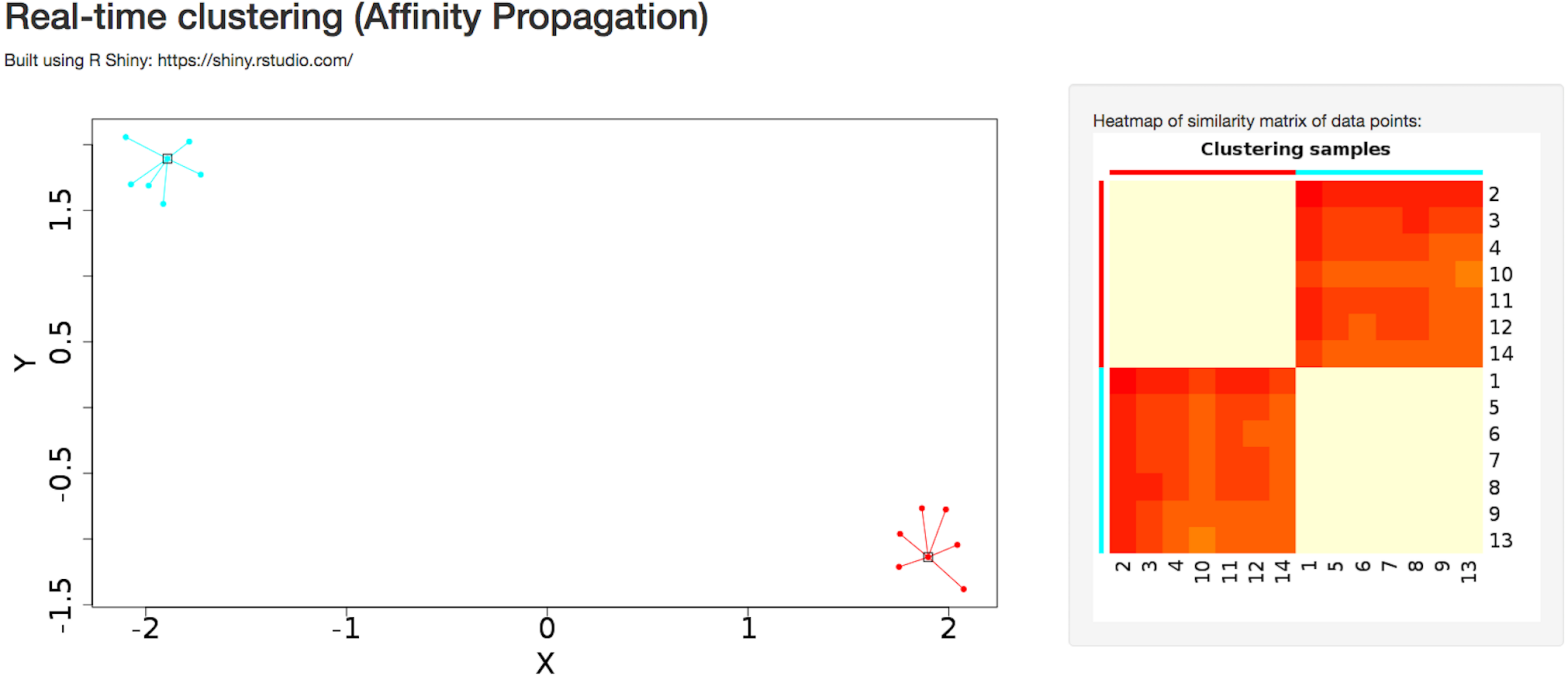
Dynamic clustering application in affinity propagation using R Shiny server displaying heatmap of similarity matrix of selected data points.

A detailed tutorial page on the website provides the user with a summary of advantages and disadvantages of each of the clustering algorithms. Interactive clustering from the R Shiny package (Chang et al., 2015) is available for affinity propagation and Gaussian mixture model, allowing the user to add data points dynamically through the GUI and observe changes in clustering behavior in real time (Fig. 4). The tutorial page further discusses pathological situations in which each algorithm may fail, with modified examples from the Scikit-learn Python package (Pedregosa et al., 2011).

### Sample data

Figure 3 shows snapshots of clustering results of NCI60 sample data using the k-medoids algorithm and 10 clusters. The samples are labeled using the same color scheme for both 2D and 3D visualizations. In Fig. 3A, the k-medoids algorithm is able to separate closely related samples in terms of gene expression. For example, in the plot corresponding to PC1-PC2, the two breast tumor samples (green color) are identified in a cluster different from the nearby melanoma samples (gray color). In a similar way, one can compare the clustering results across different algorithms and assess them based on biological knowledge.

**Figure 5:**
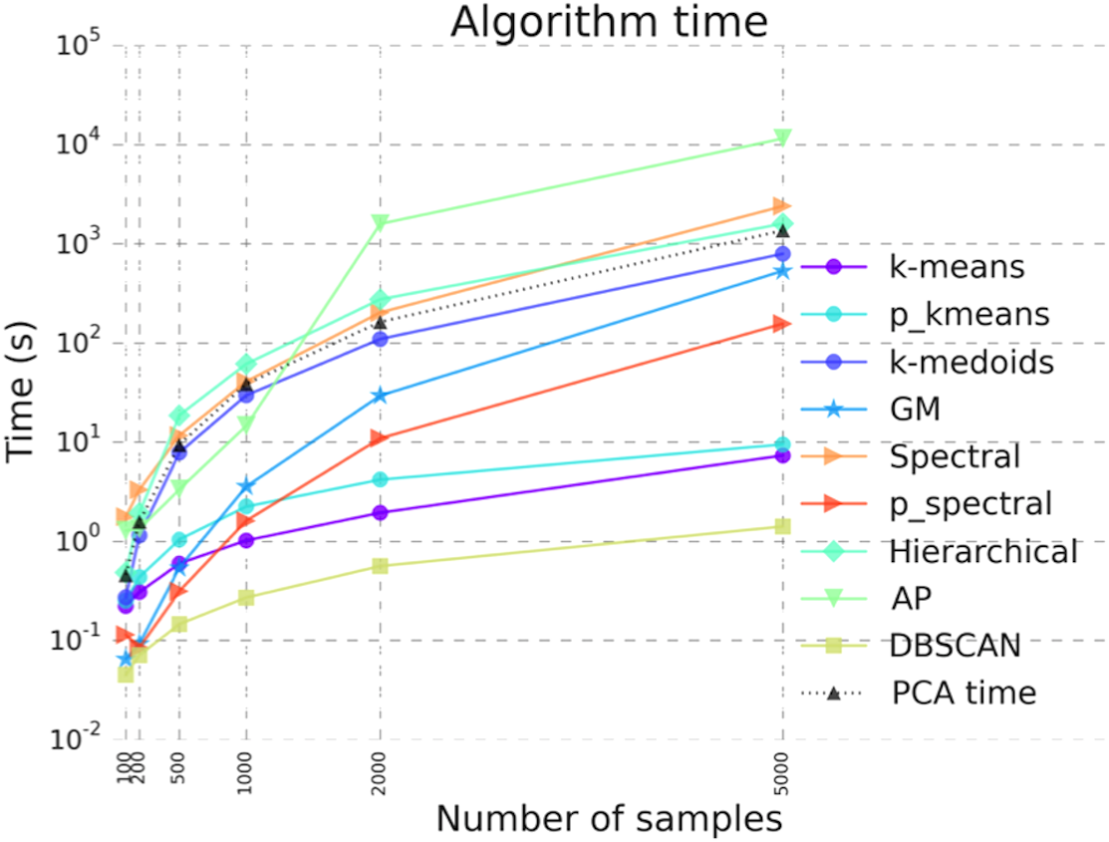
Benchmarking results illustrating algorithm run time for the clustering algorithms in ClusterEnG. “PCA time” data indicates the time taken to compute principal components, a step common to all the algorithms for visualization.

## Discussion

We have benchmarked the performance of the codes for all available clustering algorithms. Figure 5 shows the runtime of various clustering algorithms on ClusterEnG as a function of number of samples. The test data are randomly generated from five Gaussian distributions with different mean over the feature set. The number of features for each dataset is fixed at 10,000, while the number of samples is varied from 100 to 5,000. Figure 5 also includes the time taken to perform PCA on the test data. The PCA step is common to all the algorithms. As shown in Fig. 5, DBSCAN performs best with respect to runtime for all tested data, whereas affinity propagation and hierarchical clustering have maximum runtimes for larger and smaller sample sizes, respectively. However, DBSCAN and affinity propagation can give different numbers of clusters, since these algorithms estimate the number of clusters from data. In the above analysis, the parallel k-means and spectral clustering algorithms are run on a single core for comparison with serial codes. We note that the above benchmarking was performed with datasets having a similar data structure (Gaussian distribution). The actual runtime of each algorithm may vary from dataset to dataset.

For the NCI60 and B-cell lymphoma gene expression datasets, hierarchical clustering performs best in terms of the quality of clustering (with default parameters), as assessed by the number of validation measures suggesting optimal clustering. Specifically, eight and eleven of the eighteen validation measures indicated that hierarchical clustering is optimal for the NCI60 and B-cell lymphoma datasets, respectively. However, we note that the quality of a clustering result crucially depends on the geometric structure of the data being analyzed. For example, the comparison plot on the website shows that spectral clustering and DBSCAN can correctly cluster the concentric annuli data, while the other algorithms fail to identify the correct clusters.

We are currently developing and implementing parallel algorithms for affinity propagation and hierarchical clustering, and they will be included in the future releases of ClusterEnG. Furthermore, we plan to incorporate modules for exporting the clustering results directly to other available web servers for integrative analyses, including gene ontology and gene set enrichment analysis.

## Conclusions

ClusterEnG offers a one-stop web service for efficient clustering of large datasets with the flexibility of choosing among many state-of-the-art clustering algorithms, which are not readily accessible to beginners. The included interactive visualizations of clustering results in 2D and 3D will enable the users of our resource to comprehend their data effectively. We are exploring the possibility of accepting datasets much larger than the current limit by allowing the user to perform clustering on our server and then download the results for further analysis and/or visualization. As is the case for other visualization web resources, ClusterEnG’s interactive visualization module for large datasets depends on the user’s system specifications. Nevertheless, an alternative approach would be to visualize only user-selected samples after clustering.

## Additional Information and Declarations

### Data Availability

The following information was supplied regarding data availability: The R scripts used for clustering can be found at https://github.com/KnowEnG/ClusterEnG.
